# Combined Therapies for Duchenne Muscular Dystrophy to Optimize Treatment Efficacy

**DOI:** 10.3389/fgene.2018.00114

**Published:** 2018-04-10

**Authors:** Gonzalo Cordova, Elisa Negroni, Claudio Cabello-Verrugio, Vincent Mouly, Capucine Trollet

**Affiliations:** ^1^Sorbonne Université, Institut National de la Santé et de la Recherche Médicale, Association Institut de Myologie, Centre de Recherche en Myologie, UMRS974, Paris, France; ^2^Laboratorio de Patologías Musculares, Fragilidad y Envejecimiento, Departamento de Ciencias Biológicas, Facultad de Ciencias Biológicas, Universidad Andres Bello, Santiago, Chile; ^3^Millennium Institute on Immunology and Immunotherapy, Santiago, Chile

**Keywords:** gene therapy, cell therapy, muscle, Duchenne muscular dystrophy, dystrophin, fibrosis, inflammation, atrophy

## Abstract

Duchene Muscular Dystrophy (DMD) is the most frequent muscular dystrophy and one of the most severe due to the absence of the dystrophin protein. Typical pathological features include muscle weakness, muscle wasting, degeneration, and inflammation. At advanced stages DMD muscles present exacerbated extracellular matrix and fat accumulation. Recent progress in therapeutic approaches has allowed new strategies to be investigated, including pharmacological, gene-based and cell-based therapies. Gene and cell-based therapies are still limited by poor targeting and low efficiency in fibrotic dystrophic muscle, therefore it is increasingly evident that future treatments will have to include “combined therapies” to reach maximal efficiency. The scope of this mini-review is to provide an overview of the current literature on such combined therapies for DMD. By “combined therapies” we mean those that include both a therapy to correct the genetic defect and an additional one to address one of the secondary pathological features of the disease. In this mini-review, we will not provide a comprehensive view of the literature on therapies for DMD, since many such reviews already exist, but we will focus on the characteristics, efficiency, and potential of such combined therapeutic strategies that have been described so far for DMD.

## Introduction

Duchenne Muscular Dystrophy (DMD), the muscular dystrophy, is an X-linked recessive disease that affects one in 3,500 live male births (Bushby et al., 2010). DMD patients present progressive muscular weakness, in addition to orthopedic, respiratory, and cardiac complications that lead to their death around the third or fourth decade of life (McNally, 2007; Bushby et al., 2010). At the molecular level, DMD is caused by mutations in the dystrophin gene leading to the absence of the protein (Koenig et al., 1987; Kunkel et al., 1987). The dystrophin gene is one of the largest genes in the human genome with more than 2 million base pairs in Xp21.2-p21.1. The size of the dystrophin coding sequence (11 kbp) is huge with 79 exons encoding a 427 kDa protein (Guiraud et al., 2015). Most of the DMD patients carry out-of-frame and non-sense mutations leading to reduction of the transcript level and truncation of translation (Monaco et al., 1988; Roberts et al., 1994). Dystrophin is a cytoskeletal protein critical for the stability and function of myofibers in muscle: dystrophin establishes a mechanical link between the extracellular matrix and the cytoskeletal actin in muscle fibers through the dystrophin-associated protein complex (DAPC) (Ervasti and Sonnemann, 2008). Dystrophin deficiency leads to the rupture of the muscle fiber membrane during contraction (Allen and Whitehead, 2011) and causes impaired intracellular signaling (Constantin, 2014). At the cellular level, the muscles of DMD patients show evidence of necrosis, degeneration and regeneration, myofiber atrophy, fatty accumulation, fibrosis, and inflammation (Spencer and Tidball, 2001; Alvarez et al., 2002; Desguerre et al., 2009a,b; Serrano and Muñoz-Cá-noves, 2010; Zhou and Lu, 2010; Villalta et al., 2011). Different approaches (gene-based, cell-based, nano-particles, and pharmacological) have been developed to restore a functional dystrophin to DMD muscles (Negroni et al., 2016; Chamberlain and Chamberlain, 2017; Nance et al., 2017). These strategies are promising and several clinical trials are on-going or have been conducted on DMD patients: between 1995 and 2018, 127 clinical trials are found on clinicaltrials.gov, with 57% pharmacological approaches, 28% gene-based (22% antisense oligonucleotide based exon skipping, 6% AAV gene addition), and 3% cell-based approaches. To maximize the efficiency of gene- and cell-based approaches, future therapies will have to take into account the state of the muscle tissue and the secondary modifications associated to the genetic defect. For example, the integrity of the sarcolemma of muscle fibers, essential for efficient and long term gene therapy, is severely compromised in DMD (McElhanon and Bhattacharya, 2018), which leads to the concomitant loss of the therapeutic agent (Le Hir et al., 2013). In addition, in dystrophic muscle the continuous breakdown of muscle fibers causes inflammation and fibrosis (Serrano and Muñoz-Cánoves, 2017). Such a hostile environment will be detrimental for the efficacy of cell-based therapies and exacerbated extracellular matrix will affect the accessibility of all therapeutic agents to the muscle fibers (gene, cell, or pharmacological).

**Figure 1:**
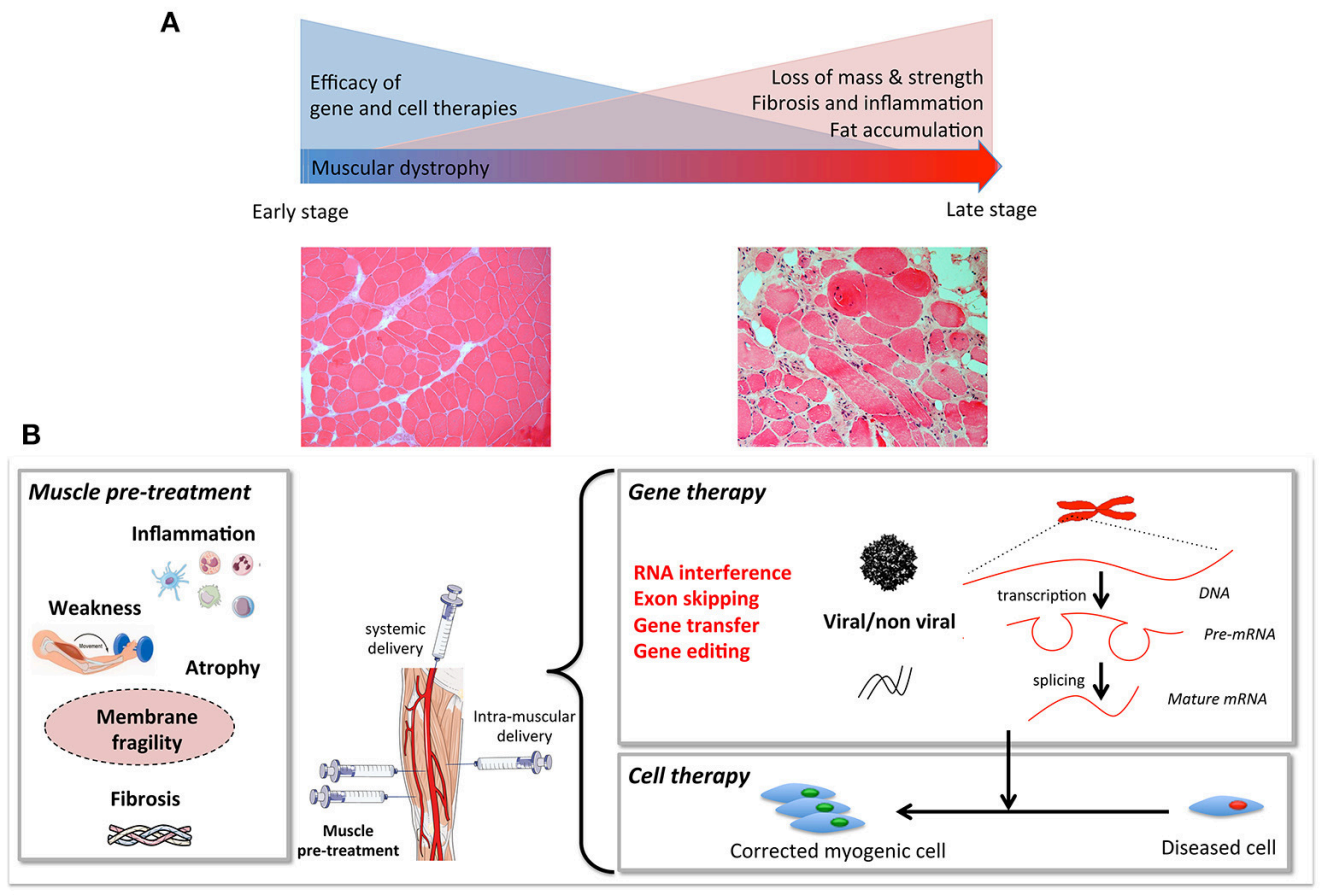
**(A)** Muscular dystrophies fibers, including loss of mass, weakness, fat, and extracellular matrix accumulation. Gene and cell based therapies will have to overcome the progressive degeneration of muscle fibers. When these histological changes become prominent, combined strategies are needed. **(B)** Muscle pre- or co-treatment may target inflammation, atrophy, membrane fragility, muscle weakness, and/or atrophy to pre-condition the tissue to increase efficiency of gene and cell therapy.

The aim of this review is to highlight pre-clinical studies in DMD that have tested two therapeutic strategies in combination (Figure 1). We will only focus on studies that have used “combined therapies”: one to correct the genetic defect, and a second to improve the status of the recipient muscle. To improve the dystrophic environment of the recipient tissue, strategies are being developed to eliminate the barriers that limit the access of the therapeutic vector to the fibers and to limit degeneration—even temporarily—to allow dystrophin to reach therapeutic expression levels. “Muscle conditioning” treatments ameliorating the status of the targeted muscle are being developed. Such improvements *per se* are beneficial to the muscle and will also improve the efficacy of gene or cell-based therapy. The final goal of any combined therapy should be to improve the efficacy of the single target therapies. Since an extensive literature on the different single target strategies developed for DMD already exist, we will not describe these here, nor shall we discuss animal models used for the same reason.

### Improving dystrophin expression using combined therapy

Exon skipping approaches have already shown promising results in animal models. This therapy is based on the use of antisense oligonucleotides (AON), that will interfere with the normal splicing process removing the mutation-carrying exons, allowing the production of a truncated but still functional dystrophin (Nakamura, 2017). Indeed the dystrophin structure with its central rod-domain made of 24 spectrin-like repeats, can tolerate large internal deletions while maintaining most of its function. In an elegant study, Peccate et al. recently demonstrated that a pre-treatment of the skeletal muscle of *mdx* mice (the most common mouse model for DMD; Bulfield et al., 1984) with peptide-phosphorodiamidate morpholino (PPMO) antisense oligonucleotides targeting dystrophin was beneficial for a subsequent AAV-based exon-skipping therapy (Peccate et al., 2016). This pre-treatment allowed temporary restoration of dystrophin at the sarcolemma, improving membrane integrity to reduce the loss of vector genome after AAV injection and improve the efficiency of gene therapy. This study emphasizes the strong potential of combined approaches to improve the benefit of AAV-based therapies since without pre-treatment the viral vector would be lost when the muscle fibers degenerate. For DMD, such pre-treatment would allow the use of lower and thus safer vector doses for a higher level of dystrophin expression in the long term. Such pre-treatment aiming at improving muscle fiber integrity could benefit also to other muscular dystrophies with degenerative features. The efficiency of exon-skipping can also be targeted. Using high-throughput screening, Kendall et al. identified Dantrolene—currently used to treat malignant hyperthermia—as a “skipping enhancer” (Kendall et al., 2012). This drug delivered to *mdx* mice by intraperitoneal injections enhanced antisense oligonucleotide (AON)-mediated DMD exon skipping. The use of such an enhancer will improve AON treatment by increasing the therapeutic value of AON, reducing the dose needed, and thus lowering the costs and potential toxicity. Finally, nanotechnologies have also been used to deliver therapeutic agents, such as antisense nucleotides (for a review see Falzarano et al., 2014). Such tools might in the future be used in combined therapeutic strategies.

### Stimulation of muscle growth and regeneration

If muscle wasting has already progressed, dystrophin expression in the surviving fibers will not be sufficient to restore function. Maintaining and stimulating higher levels of muscle regeneration could potentially have a beneficial effect in dystrophic muscles. The first attempt of a combined therapy stimulating muscle growth came from Abmayr et al. (2005), who used the co-expression of Insulin-like Growth factor-1 (IGF-1)—a known inducer of muscle hypertrophy, strength and regeneration (Philippou and Barton, 2014)—together with the expression of a functional microdystrophin (μDys) in *mdx* mice. Muscles treated with this combined therapy, showed increased muscle mass and specific force compared to untreated or to muscles treated with μDys alone. A similar approach was used by Rodino-Klapac et al. (2013) by combining follistatin—an inhibitor of myostatin (Sharma et al., 2015)—to increase muscle mass and strength, and μDys. They showed a potent synergistic effect of the combined therapy on muscle force and architecture. This was also demonstrated with an AON triggering exon skipping of dystrophin and another one targeting myostatin to improve muscle weakness (Kemaladewi et al., 2011; Lu-Nguyen et al., 2017) with promising results. Similar approaches to interfere with the myostatin pathways have also been used in combined therapies: RNA interference for the Activin Receptor type IIb (AcvRIIB) (Dumonceaux et al., 2010)—the receptor for Myostatin—or a soluble version of AcvRIIB (Hoogaars et al., 2012) have been used in combination with AAV-U7 based exon skipping resulting in a beneficial effect increasing both muscle mass and strength.

### Controlling fibrosis, inflammation, and atrophy

Fibrosis, inflammation and muscle atrophy are among the most important complications associated with muscular dystrophy, and they can severely compromise the efficiency of gene or cell therapy by limiting access to the dystrophic muscle. Fibrosis can be defined as the increased expression and accumulation of Extracellular Matrix (ECM) proteins, such as fibronectin and collagen, which contributes to muscle dysfunction (Serrano and Muñoz-Cánoves, 2017). Transforming Growth Factor type β (TGF-β) is a potent pro-fibrotic cytokine that contributes to the pathogenesis of several fibrotic disorders, including muscular dystrophies (Bernasconi et al., 1999). Interestingly, it has been found that TGF-β induces the expression of Connective Tissue Growth Factor (CTGF/CCN2) in fibroblasts (Igarashi et al., 1993) and the pro-fibrotic effects of TGF-β may be CTGF-dependent (Grotendorst, 1997; Leask and Abraham, 2004; Leask et al., 2004). The expression of microRNA-29—a family of microRNAs whose downregulation is associated with fibrosis—not only decreased TGF-β1 and ECM proteins expression but also completely restored muscle strength in dystrophic muscle when combined with μDys treatment (Heller et al., 2017). Similarly, reducing CTGF expression genetically or blocking CTGF with neutralizing antibodies, decreased fibrosis, and increased muscle strength and the efficiency of cell therapy (Morales et al., 2013b). Combining different cell types in cell therapy has also been shown to improve the fibrotic environment in dystrophic mice. Gargioli et al. showed that a pre-treatment using modified tendon fibroblasts, expressing angiogenic factors such as placenta growth factor and an antifibrotic treatment using MMP-9, improved microcirculation, reduced collagen and fat tissue deposition, decreased leukocyte infiltration, increased fiber numbers and improved cell-therapy in aged α-Sarcoglycan null mice, a model of Limb-girdle muscular dystrophy (Gargioli et al., 2008).

Inflammation is part of the normal regeneration process where macrophages play a fundamental role in both inflammation and regeneration by the sequential expression of cytokines and inflammatory molecules (Juban and Chazaud, 2017). It is now well-established that the inflammatory response in damaged muscle positively influences normal muscle repair, while its exacerbation in dystrophic muscle promotes the formation of fibrotic tissue during disease progression (Tidball, 2005). For these reasons, targeting inflammation may improve therapies for DMD (Miyatake et al., 2016). Several different combined approaches have been used to decrease inflammation and improve therapeutic outcome: anti-inflammatory prednisolone combined with AON exon skipping treatment has been shown to increase dystrophin expression (Verhaart et al., 2012). A study by Cabrera et al. (2014) showed that andrographolide—an inhibitor of NF-κB (pro-inflammatory pathway implicated in atrophy and fibrosis; Li et al., 2008)—reduces the expression of fibrotic factors and ECM proteins, while increasing muscle strength and cell therapy efficacy. Another study showed that treatment with HCT 1026—a non-steroidal anti-inflammatory drug capable of releasing nitric oxide (NO)—increased the efficiency of cell therapy in *mdx* mice and in a mouse model of limb girdle muscular dystrophy (Brunelli et al., 2007). NO deficiency in DMD, due to the disappearance of nNOS linked to the dystrophin complex, is also an important issue since it is a potent regulator of skeletal muscle physiology and regeneration, and could also be targeted in combined therapies (Timpani et al., 2017).

Muscular atrophy is a common feature of DMD and many pathological processes discussed in this mini-review contribute to muscle wasting (Shin et al., 2013). One of the pathways involved in the regulation of muscle mass is the Renin-Angiotensin System (RAS) (Cabello-Verrugio et al., 2012a, 2015). Several of its components are upregulated in dystrophic muscles (Sun et al., 2009) where they can also trigger a fibrotic response. Pharmacological modulation of RAS can be used to decrease atrophy (Burks et al., 2011), decrease fibrosis (Cabello-Verrugio et al., 2012b; Morales et al., 2013a; Acuna et al., 2014) and ameliorate cardiac complications related to MD (Allen et al., 2013; Sabharwal et al., 2014). Several studies have used combined therapies using Losartan, an inhibitor or the AT-1 receptor. Losartan treatment has been shown to increase the efficiency of myoblast cell therapy (Fakhfakh et al., 2012) and Adipose-Derived Stem Cell therapy (Lee et al., 2015). However, a study by Lee et al. (2014) showed that although combined therapy with Losartan and exon skipping was beneficial in terms of muscle regeneration, the efficiency of exon skipping was lower in Losartan treated mice due to decreased *in vivo* morpholino penetration. In this study, Losartan was added prior to morpholino treatment, it would be interesting to see what happens when losartan treatment is started after the exon skipping treatment. Moreover, as Losartan seems to increase sarcolemma stability, it could be interesting to see the effect of viral gene therapy after Losartan treatment.

### *In vitro* modification of myogenic cells

Among “combined therapies” those combining gene- and cell- therapies should be mentioned, even though none of the two strategies improve the status of the recipient muscle. Most combined gene- and cell-based studies developed so far consist in genetic modification of adult stem cells harvested from patients or dystrophic models to produce a functional dystrophin protein. In order to accommodate DNA packaging limitations in a range of viral vectors, synthetic mini- and micro-dystrophin versions have been engineered (Athanasopoulos et al., 2004) and tested in cell transplantation studies using lentiviral vectors. Several transduced types of myogenic cells, e.g., mouse, canine, primate, and human muscle precursors (Ikemoto et al., 2007; Quenneville et al., 2007b; Pichavant et al., 2010), murine side population (SP) cells (Bachrach et al., 2004), canine (Sampaolesi et al., 2006), and human (Dellavalle et al., 2007) mesoangioblasts/pericytes, have been tested in dystrophic models. The group of J. Tremblay demonstrated that the use of electroporation combined with the introduction of a phiC31 integrase led to the stable expression of full-length dystrophin in murine and human MPCs, even if this technique is less efficient than viral vector transduction (Quenneville et al., 2007a). Kazuki et al. have also validated the use of a human artificial chromosome (HAC) to restore full-length dystrophin in mouse and human iPS cells (Kazuki et al., 2010), while genomic integration of the full-length human dystrophin has been achieved in iPS cells (Farruggio et al., 2017) and mesangioblasts (Loperfido et al., 2016). Also, a full-length dystrophin was efficiently expressed in dog mesoangioblasts using piggyBac transposons (Loperfido et al., 2016). The same technique was used to modify mouse mesoangioblasts prior to transplantation in *mdx* mice, showing a good level of dystrophin expression, increased number of satellite cells, reduction in fibrosis, and increased muscle function (Iyer et al., 2018). Exon skipping has also been tested in combination with cell therapy approaches using targeted antisense sequences vectorised in U7 snRNA constructs in skin fibroblasts (Chaouch et al., 2009) or CD133+ cells of DMD patients (Benchaouir et al., 2007).

More recently, direct targeting of a morbid allele has been challenged using nucleases *in vitro* and *in vivo:* meganucleases, Zinc-finger nucleases, TALENs, and CRISPR have all been used for genome editing to correct DMD cells carrying deletions and out-of-frame mutations in dystrophin gene (Ousterout et al., 2013, 2015b; Popplewell et al., 2013; Young et al., 2016; Gee et al., 2017; Pini et al., 2017; Reinig et al., 2017; Wang et al., 2017; Zhu et al., 2017). A recent study also described a multiplexed strategy using a lentiviral vector capable of editing multiple sequences at a time, allowing the correction of up to 62% of mutations causing DMD (Ousterout et al., 2015a). While none of these approaches have yet been used to our knowledge in combined therapies, they could also profit from such strategies.

## Concluding remarks

### Combined therapies for DMD

There is now an increased interest in developing combined therapies for DMD (Table 1). A combined therapy is most often designed to treat the secondary consequences of the muscular dystrophy that decrease the efficiency of single therapies, e.g., inflammation, fibrosis, or degeneration. If efficient, this therapy should have two effects: (1) improve the muscle phenotype *per se* and, (2) improve the combined therapy by pre- or co-conditioning the muscle that is receiving the treatment. In other words, combined therapies should have a synergistic effect. It is essential to combine the positive outcomes of distinct therapies that target these different features to enhance therapeutic efficiency. Many combinatory studies could be tested, just to name one for example, it would be interesting to know if SMT-C1100—an utrophin upregulation drug (Tinsley et al., 2011), already tested in clinical trials with promising results and no side-effects (Ricotti et al., 2016)—shows a synergistic effect when combined with other therapies, like cell therapy.

**Table 1:** Overview of synergistic therapies tested in muscular dystrophies.

**Therapy #1**	**AAV**	**Antisense**	**Therapy #2**	**Viral vectors**	**Antisense**	**Pharmacological**	**Muscle mass and/or strength**	**Fibrosis and inflammation**	**Pre-treatment**	**Co-treatment**	***In vitro* model**	**Benefit**	**References**
Cell therapy			NO/HCT 1026			X	X		X		X	Increased mesoangioblast cell-therapy, muscle force, and animal performance in exhaustion treadmill tests. Reduced CK activity in serum in α-SG null mice.	Brunelli et al., 2007
Cell therapy			PIGF/MMP9	X				X	X		X	Reduced collagen. Increased mesoangioblast cell-therapy and increased number of fiber per CSA in α-SG null mice.	Gargioli et al., 2008
Cell therapy			Losartan			X		X	X	X	X	Reduced collagen expression. Increased number of fibers and nuclei from transplanted human myoblasts and increased survival after transplantation in Rag^−/−^/mdx mice.	Fakhfakh et al., 2012
Cell therapy			CTGF/CCN2 genetic reduction and blockage			X		X	X			Increased number of Dystrophin fibers after mouse myoblast cell-therapy and decreases fibrosis in mdx and mdx-CTGF^+/−^ mice.	Morales et al., 2013b
Cell therapy			Andrographolide			X	X	X	X			Reduction of fibrosis. Increased muscle force and cell-therapy with wt mouse satellite cells in mdx mice.	Cabrera et al., 2014
Cell therapy			Losartan			X	X	X		X	X	Reduction of fibrosis. Increased effect in ADSC cell-therapy, increased muscle weight and increased fibers in mdx mice.	Lee et al., 2015
Exon skipping	X		sh-ActRIIb		X		X					Increased muscle weight and force, and increased fiber diameter in mdx mice.	Dumonceaux et al., 2010
Exon skipping		X	Myostatin		X						X	Dual myostatin and dystrophin skipping *in vitro* in human control and DMD cells, and in the mdx mice.	Kemaladewi et al., 2011
Exon skipping	X		sActRIIB-Fc			X	X			X		Improved muscle strength and dystrophin rescue, no evidence of synergistic effect in the mdx mice.	Hoogaars et al., 2012
Exon skipping		X	Dantrolene			X	X			X	X	Increased exon skipping, decreased CK levels, and improve muscle strength in mdx mice.	Kendall et al., 2012
Exon skipping		X	Prednisolone			X				X	X	Increased Dystrophin expression in gastrocnemius of mdx mice.	Verhaart et al., 2012
Exon skipping		X	Losartan			X		X	X	X		Increased muscle regeneration and reduction of dystrophin expression in mdx mice.	Lee et al., 2014
Exon skipping	X		AON Dystrophin		X		X		X			Decrease gene therapy vector loss, increase dystrophin expression in mdx mice.	Peccate et al., 2016
Exon skipping		X	Myostatin		X		X	X				Increased exon skipping and dystrophin expression, decreased fibers with central nuclei, decreased collagen VI, increased muscle strength and improves animal behavior in mdx mice	Lu-Nguyen et al., 2017
Microdystrophin	X		IGF-1	X			X					Increased muscle mass and strength, reduced myofiber degeneration, and reduced contraction-induced injury in mdx mice.	Abmayr et al., 2005
Microdystrophin	X		Follistatin	X			X					Increased muscle force and resistance to injury, restored fiber size in young and old mdx mice.	Rodino-Klapac et al., 2013
Microdystrophin	X		MicroRNA-29 overexpression	X			X	X		X	X	Reduced fibrosis, increased muscle strength, reduced contraction-induced injury, increased muscle size in mdx/utrn^+/−^ mice.	Heller et al., 2017

*a-SG, alpha-sarcoglycan; DMD, Duchenne Muscular Dystrophy; MDC1A, congenital muscular dystrophy type 1A; AAV, adeno-associated virus; AON, antisense oligonucleotide; NO, Nitric oxide*.

*Pink, type of treatment (viral vectors, antsiense, or pharmacological)*.

*Blue, status of the muscle (muscle mass and/or strength, fibrosis, and inflammation)*.

*Green, whether this is a pre or a co treatment*.

*Orange, whether this was done in in vitro model*.

Of course, the advances in combined therapies should not stop the efforts that have been conducted to ameliorate single target therapies for DMD since they will eventually also benefit to these combined therapies. Future technical advances in distinct approaches will help to improve combined therapeutic assays that will eventually lead to the effective treatment or even a cure for DMD.

### Early detection and treatment vs. symptomatic patients

It is important to make this distinction. The early detection of dystrophin deficiency and a precise genetic diagnosis (Aartsma-Rus et al., 2016) will allow the treatment to be started before the onset of fibrosis, chronic damage, and inflammation in the muscle. A genetic correction might then be enough to avoid the progression of the disease. With this in mind, a screening test for the presence of a fully functional dystrophin for all male newborns could potentially result in an invaluable social and monetary benefit for the families and the health care system (Landfeldt et al., 2016; Ryder et al., 2017). However, the diagnosis for sporadic mutations is usually done when the patients start to show their first symptoms in early childhood and, at this moment, muscles already show extensive damage, inflammation and fibrosis. In this case, secondary effects of the gene deficiency should be addressed in combined therapies to enhance the correction of the genetic defect.

## Author contributions

All authors listed have made a substantial, direct, and intellectual contribution to the work, and approved it for publication.

### Conflict of interest statement

The authors declare that the research was conducted in the absence of any commercial or financial relationships that could be construed as a potential conflict of interest.
